# Fractures of the Malar Bone, Superior Maxillary, Ethmoid, and of Other Bones of the Face

**Published:** 1857-10

**Authors:** Frank H. Hamilton


### BUFFALO MEDICAL JOURNAL

AND

### MONTHLY REVIEW.

**VOL. 13.** OCTOBER, 1857. **NO. 5.**

### ORIGINAL COMMUNICATIONS.

ART. I.—*Fractures of the Malar Bone, Superior Maxillary, Ethmoid, and of other bones of the Face.* By FRANK H. HAMILTON, M. D.

### MALAR BONE.

I have been unable to find any records of a simple fracture of the malar bone, that is to say, of a fracture unconnected with a fracture of other bones of the face. It is probable, however, that it sometimes occurs, but that not being accompanied with much displacement, it is overlooked. I have myself seen a fracture of the upper margin, or of that portion which constitutes a part of the orbital border, in two or three instances, while I was unable to detect any other fracture among the bones of the face; but it is by no means certain that other fractures did not exist, perhaps in some of the bones which form the socket, or in the superior maxilla, as mere fissures, or as fractures with only slight displacement. The prominence of the malar bone and especially the sharpness of this orbital margin would enable the surgeon to detect easily the smallest displacement, or even a fissure, while a much more extensive displacement elsewhere would escape detection.

The following observations will illustrate these remarks:

*Observation First.* With a heavy steel hammer I struck the left malar bone of a naked skull breaking the orbital margin near its middle, the line of fracture extending backward through the whole length of the orbital plate, and the outer half of the plate being pressed about two lines into the orbital cavity. There was no other fracture of the malar bone.

The nasal process of the superior maxilla was broken near its base, and the whole upper portion of this bone was thrown inward toward the nasal passages in such a manner as nearly to close them; while its lower margin was thrown out, separating the two upper maxillary bones from each other along the whole line of the inter-maxillary suture. This separation was about two lines in front and less toward the palatal bones.

The ethmoid was fissured through the whole length of its os planum, from before backward.

*Remarks.*—It is very easy to understand how a blow upon the malar bone, coming a little from one side, should produce this form of displacement of the superior maxilla.

The two upper maxillary bones form, as they are placed opposite to each other, an irregular arch, one end of which rests upon its fellow, at the inter-maxillary suture, and the other end rests upon the nasal and frontal bones; while over the centre of the arch is situated the malar bone. The force of a side blow upon the malar bone will expend itself therefore chiefly upon the base of the maxillary apophysis, as being in the line of the direction of the force. The force continuing to act, after the apophysis is broken, the portion of the superior maxilla above the floor of the nares will fall inward toward the septum, while the portion below will tilt outward and open the inter-maxillary suture along the roof of the mouth. This suture will also open more widely in front than behind, owing to the greater depth of the suture in front.

One might suppose that it would be a very easy matter to restore these bones to place upon the naked skull, after such an accident. Certainly it would be very desirable to do so, were this accident to occur to any patient, since the malar bone is slightly depressed, the nostril upon this side is nearly closed, and the line of the teeth is disturbed, and it is possible also that an opening might be established between the nose and mouth immediately back of the incisors. In fact, however, I found the restoration impossible. It could not be accomplished by an instrument within the nose pressing outward, nor by pressing inward upon the teeth and alveola; not certainly without very great and unwarrantable force. The difficulty consisted simply in the antagonisms of the serrated margins of the inter-maxillary suture, which projecting one or two lines on each side, could not be made to interlock again, but were firmly braced against each other.

A repetition of the blow broke the malar bone completely in two, disarticulating it also at its zygomatic suture.

The superior maxilla was now separated wholly from the other bones of the face and skull, carrying the palate bone with it entire. The body and sinus of the upper maxilla still, however, remained unbroken.

*Observation Second.* Blow inflicted with the hammer, square upon the malar bone, broke the malar bone on its orbital margin in the same manner as in observation first. There was also a fissure on the upper and outer margin of the bone. There was no other fracture of the malar bone.

The zygoma was broken transversely near its middle, through that portion which belongs to the temporal bone.

The superior maxilla was broken through the antrum, a little below the base of the malar eminence, and the malar bone was forced into the antrum several lines. The alveola was not broken.

The ethmoid bone was fissured antero-posteriorly through its orbital plate.

*Remarks.* In this example the walls of the antrum having at once given way, the force of the blow did not reach the nasal apophysis. It was found very easy to lift the malar bone to its place, with an instrument introduced through the broken walls of the antrum.

*Observation Third.* The hammer falling upon the malar bone depressed it slightly, but did not break it.

The superior maxilla was found broken through the walls of the antrum a little below the malar apophysis. No other fracture.

The *second* blow broke the malar bone through its centre, breaking also the zygoma near its centre.

The *third* blow broke the nasal apophysis of the superior maxilla near its base, comminuting the antrum; into which the malar bone was forcibly driven. At the same time the orbital plate of the malar bone was thrown up into the socket. The ethmoid was also broken.

*Observation Fourth.* *First* blow did not fracture the malar bone, but broke the walls of the antrum, and at the same moment produced a fissure extending vertically through the alveola between the first, and second molars, into the mouth.

The *second* blow broke the malar bone through its centre irregularly, and also the zygoma transversely, near the centre of its arch. The nasal process of the superior maxilla was broken at two points, one of the lines of fracture extending into the orbital socket.

The skull was also found broken at its base through the lesser wings of Ingrassins. The force of the blow having been conveyed, apparently, along the orbital plate of the superior maxilla and os planum.

*Remarks.* This is the only example in which the fracture has extended through the alveola, and it was the result of the first blow. The fracture of the base of the skull by the second blow indicates the possibility of producing a fatal lesion of the brain or of its blood-vessels by a blow upon the malar bone.

*General Summary.* A fracture of the superior maxilla has occurred in every instance: and twice when the malar bone was not broken; in each of the two last cases the antrum alone was broken and the depression of the malar bone was scarcely noticeable. In the second of these cases, the fracture extended also through the alveola.

In three cases the nasal apophysis has broken near the base, and in one case at two points. One of the three fractures of the nasal apophysis was accompanied with a diastasis of the superior maxilla through its inter-maxillary suture.

The malar bone has been broken twice by the first blow, and always when the blow has been repeated. The orbital margin and orbital plate has been fissured twice, the outer portion of the orbital plate being pushed a little into the socket. Once this plate has been pushed downward.

The zygoma has been broken three times, and always transversely, a little beyond its centre, or where the bone is the most slender and most convex.

The ethmoid has been been broken three times, and always longitudinally through the orbital plate.

The sphenoid has been broken once, at the base of the skull.

In addition to these observations upon the naked skull, I have seen two examples, which illustrate the relative infrequency of fractures of the malar bone, as compared with fractures of the superior maxilla and of the other bones of the face, even when the blow is received directly upon the malar bone.

Case I. Pat Maloney, æt. 55, fell about twenty feet and struck upon his face. Six weeks after the accident, while an inmate of the Buffalo Hospital of the Sisters of Charity, I found the right malar bone depressed, but I could not trace any line of fracture in the malar bone. I think the antrum of the superior maxilla was broken and the malar bone forced in upon it.

Case II. Thomas Crotty, æt. 20, was struck with a hoop, August 15, 1855. He was seen immediately by a surgeon in Canada, but the fracture was not recognized. Five days after he called at my office. I found the outer portion of the right malar bone lifted slightly and the lower and anterior angle depressed about three lines, as if this portion had been forced in upon the antrum.

*Prognosis.* The malar bone may be depressed, as we have seen, to the extent of two or three lines, without being broken. This accident will be more properly considered under fractures of the upper maxilla. A fracture of the malar bone implies, therefore, generally, that great force has been applied, and that other fractures exist as complications. This may not be true, however, when only the orbital margin of the socket is broken. If the orbital plate is broken, and a portion of it is pushed into the socket, it may occasion a slight protrusion of the ball, as in two cases related by Dr. Neill as fractures of the upper maxilla, and as has been noticed in the experiments already recorded. This protrusion of the eye-ball will probably continue in some degree, as long as the bones remain displaced. It is quite probable, however, that in some cases, after severe injuries of the face, a moderate protrusion of the eye-ball is due entirely to extravasation of blood in the socket; a circumstance which would be likely to follow a fracture of the bones of the socket, and to increase temporarily the protrusion of the eye.

If the body of the bone is broken entirely through, and coma supervenes upon the accident, there is some reason to fear that the skull is fractured at its base, and the prognosis ought to be grave.

*Treatment.* If there is only a fissure of the orbital margin, it will not require attention; but if the fissure extends through the orbital plate and at the same time the anterior and inferior margin of the bone is depressed, in consequence of which the orbital plate is tilted upward and made to push forward the eye-ball, the propriety of surgical interference may be considered. If this protrusion is considerable and evidently due to the displaced bone, an attempt should be made to lift the body of the malar bone and thus to restore to position its orbital plate. The method of accomplishing this I shall describe particularly when speaking of fractures of the superior maxilla with depression of the malar bones.

### UPPER MAXILLARY BONES.

These fractures assume so great a variety in respect of form, situation and complications, that it would be impossible to speak of them systematically or to establish any thing but very general rules as to treatment and prognosis.

They may be broken, or loosened from each other or from the other bones with which they are articulated, with or without any farther fracture; the nasal processes may be broken, and generally this accident is accompanied with a fracture of the nasal bones also; the malar bones may be forced in, carrying with them a portion of the outer wall of the antrum; the alveola may be broken and more or less completely detached; and either of these several fractures may be complicated with fractures of the other bones of the face or of the base of the skull even.

*Treatment.* When the harmonies of the upper maxillary bones are only slightly disturbed, nothing but a retentive treatment is necessary.

A man was thrown backward from a loaded cart, one wheel of the cart passing over his face. He was taken up unconscious, but when I saw him on the following morning, his consciousness had returned. The right malar bone was broken and forced down upon the antrum about three lines. Both superior maxillæ were loosened from their articulations, and could be moved laterally, the motion producing a slight grating sound. The same motion and grating occurred whenever he attempted to swallow. No effort was made to elevate the malar bones, nor did I find any means necessary to retain the maxillary bones in place, the amount of displacement being very inconsiderable, and never sufficient to be observed by the eye. Cool lotions were applied constantly to the face, and the patient was sustained by a liquid diet. On the ninth day all motion of the fragments had ceased, and on the twenty-seventh day the patient was completely recovered, with only the depression of the malar bone remaining.

Sargent, of Boston, reports a similar case, in which a slight separation of the maxillary bones united promptly and without any retentive apparatus.*

But in a case in which the superior maxillary bones had been more completely torn from their connections, complicated with other severe injuries, I found it necessary to support the fragments by closing the lower jaw upon the upper, and by suitable bandages. The patient died, however, on the twelfth day.†

Graefe recommends, where the bones are thus extensively separated and displaced, an apparatus made of steel, and suitably covered, which is to be applied against the forehead and buckled under the occiput. From the two sides descend a couple of steel plates, which, having arrived at the free border of the upper lip, are reflected upon themselves, and are made to support upon their extremities long silver gutters, intended for the reception of not only the displaced teeth and alveola, but also those teeth which are firm.‡

Wiseman having been summoned to a child with his whole upper jaw forced in, by the kick of a horse, "beating the ethmoides quite in from the os cribriform," and forcing the palate bone against the back of the pharynx, found great difficulty in securing a permanent reädjustment. At first he attempted to introduce his finger back of the bone, but failing in this he bent an instrument into the form of a hook, and passing it between the bone and the pharynx, he easily replaced the fragments. But on removing the instrument they were again displaced. Immediately he had constructed an instrument by which the bones could be not only easily reduced, but also retained in place, extension being made by the hands of the child, his mother and others, alternately. In this way the reünion was finally effected, and "the face restored to a good shape, better than could have been hoped for."‖

Harris, of New York, mentions a case in which a child, two years old, having fallen from a height of fifty feet upon the pavement, was found to have a diastasis of both the superior maxillary and palate bones; the separation being sufficient to admit the little finger, and extending from between the alveoli which supported the central incisors, to the soft palate. It is not said whether any efforts were made to reduce the bones, but six weeks after

---

* Boston Med. and Surg. Jour., vol. 52, p. 378.

† Report on Deformities after Fractures. Trans. Amer. Med. Association, vol. 8, p. 375, Case iv.

‡ Traité des Frac., etc., par L. F. Malgaigne, p. 373.

‖ Chirurgical Treatises, by Richard Wiseman. 1734. p. 443.

the injury was received, they were still open, and it was proposed to close the space by a plastic operation as soon as the condition of the patient would warrant such a procedure.*

I suspect that in this example, as in the first experiment quoted under fracture of the malar bone, it was found impossible to adjust the bones and close the inter-maxillary suture, and for the same reasons.

If, in consequence of a blow received upon the ossa nasi, the nasal processes of the superior maxilla are broken down, they may be lifted and adjusted in the same manner as the ossa nasi.

I have seen several examples of this accident, and I have in my cabinet a specimen, in which the nasal bones being driven in by the kick of a horse, the nasal process upon the left side is broken off just above the root of the cuspid tooth, and its upper end inclined inward toward the nasal passage and backward, until it is completely buried. In this situation it has become firmly united to the bony and soft tissues into which it was brought in contact.

The following example will illustrate some of the complications and difficulties connected with a depression of the malar bone, and consequent fracture of the antrum maxillare.

M. P., of Colesville, aged about 34 years, was thrown from a height, striking upon his face, and forcing the right malar bone down upon the antrum of the superior maxilla. Dr. L. Potter, of Varysburg, and myself were called.

The deformity produced by the sinking of the malar bone was very striking, and the patient, as well as ourselves, was very anxious to have it remedied if possible. We found some of the teeth upon the side of the fracture loose, and we determined to extract them and press up the bone with an instrument introduced through the empty sockets. The first attempt to extract a molar tooth, however, brought down several teeth, and the whole floor of the antrum. The detachment of this fragment was also now so complete that we believed it necessary to remove it entirely, a labor which was accomplished with infinite difficulty, and with no little hazard to the patient, as dissection had to be extended very far back into the throat, and in the end it was not effected without bringing out, attached to the fragment of maxillary bone, a considerable portion of the pyramidal process of the os palati.

The time occupied in this operation was at least one hour, during which we were every moment in the most painful apprehensions lest we should

---

* New York Jour. Med., vol. 13, 2d ser., p. 214.

reach and wound the internal carotid, which lay in such close juxtaposition to our knife that we could distinctly feel its pulsation. After its removal, the hæmorrhage was for an hour or more quite profuse, and could only be restrained by sponge compresses pressed firmly back into the mouth and antrum.

When the hæmorrhage was sufficiently controlled, we proceeded to examine the antrum, the floor of which being removed entire, permitted the finger to enter freely. The restoration of the malar bone was now accomplished without much difficulty, and with only moderate force.

Two years after the accident, the face presented, externally, no traces of the original injury. The malar bone seemed to be as prominent as upon the opposite side, and there was no perceptible falling in where the teeth and alveoli were removed. During several months after the removal of the bone, the antrum continued to discharge pus, but at length a semi-cartilaginous production closed in the cavity below, entirely reconstructing its floor, and the discharge ceased. Since then he has experienced no further inconvenience.

I wish to propose two or three expedients for lifting the malar bone when it has been thrust down, which may in certain cases be substituted for the mode which has been heretofore generally adopted.

In many instances, no difficulty will be experienced in resorting to the usual method. The recent loss of one or more teeth opposite the floor of the broken antrum, or the complete displacement of a tooth by the accident itself, will give an opportunity for the perforation of the antrum through the open socket, and for the introduction of a suitable instrument for lifting the depressed bone. Unless, however, the opening is quite large, the instrument employed must be so small, such as a straight steel sound or a female catheter, as to expose the parts against which its end is made to press, to some risk of being broken and penetrated. It is even possible in this way to penetrate the socket of the eye, and thus inflict serious injury upon the eye itself. Yet, with some care, such accideuts may be avoided, and it is probable that, in the cases supposed, where the sockets of the teeth opposite the base of the antrum are open, this method will continue to have the preference.

But if the teeth remain firm in their places, or if they have been some time removed, and the sockets are filled up, and we wish to enter the antrum at its base, we must either drill through its anterior wall above the roots of the teeth, or we must proceed to extract a tooth. The first method gives an inconvenient opening, and one through which it will be necessary to use a curved instrument; but yet it is a method far less objectionable than the extraction of a tooth which is firm, or which is even tolerably firm in its socket, and which may require the forceps for its removal. The objections to this latter procedure were suggested by the tedious and painful operation already detailed. The first attempt to extract a tooth brought down the whole floor of the antrum, with all its corresponding teeth and the pyramidal process of the palate bone. The tooth was already loose, and we thought it might be easily taken out, but it had not occurred to us that it was loosened by the comminuted condition of the walls of the antrum, and of the alveola. The experiments made upon the dead subject would seem to show that this fracture and comminution of the alveola is not a very frequent result of a fracture of the antrum produced by a blow upon the malar bone, yet it may happen, and whenever it does the attempt to extract a tooth must always expose the patient to the same hazards. Certainly it is no trifling matter to pull away all of a man's upper teeth upon one side, and to open freely into a broad cavity which might never close again, and which, in this event, must always serve as a place of lodgment for particles of food, and for foul secretions, to say nothing of the external deformity which it is likely to produce, and of the severity and even danger of the operation.

I wish then to suggest certain procedures, the value of which I have not yet had an opportunity to determine by any experiment upon the living subject, but which I have carefully and frequently tested upon the dead.

First, we ought to attempt to lift the bone by putting the thumb under its zygomatic process and body within the mouth. If the bone is thrown directly downward, or downward and backward, this method can scarcely fail; and even when it is thrown downward and forward so as to press into the antrum, it is likely to succeed. If, however, for any reason, the thumb cannot be brought to bear upon its under surface, we may make a small incision upon the cheek over the anterior margin of the masseter muscle, where its insertion into the malar bone terminates, and pushing a strong blunt hook under the bone, we may lift it with ease.

Where the depression of the malar bone is in the direction of the anterior and superior angle these means may not be found available, and we may then employ a screw levator, an instrument which I find already constructed in a case of trephining instruments made for me by Mr. Luer, of Paris, and which I have often used and constantly recommended to my pupils, in certain cases of fractures of the skull. The instrument ought to be made of the best steel, and with a broad, sharp-cutting thread. A slight incision being made through the skin, and down to the centre of the malar bone, the elevator is then screwed firmly into its structure, and now its elevation and adjustment may be accomplished with the greatest ease.

Malgaigne remarks: "In all complicated fractures of the upper jaw, there is one principle which surgeons cannot too much study, namely, that all fragments, however slightly adherent they may be, ought to be most carefully preserved, and they will be found to unite with wonderful ease. This remark had already been made by Saviard. Larrey insists strongly upon it and we have seen that M. Baudens, so great an advocate for the removal of loose fragments, has declared for these fractures a special exemption."*

Malgaigne has here especial reference to fractures of the alveola or to fractures implicating the alveola and extending more or less into the body of the bone.

It would be an error, however, to suppose that a reünion will in these cases uniformly take place. Exceptions have occurred in my own practice; the fragments becoming loosened and completely detached after the lapse of several weeks. In the case related by Miller, the whole floor of the antrum having been broken off, in an unskillful attempt to extract the second right upper molar, it was found impossible to make it unite, and it was subsequently removed.† Such unfortunate results certainly may sometimes be reasonably anticipated. Yet they occur so seldom as to justify the opinions and practice advocated by Malgaigne.

In some instances, where fragments are displaced carrying with them several teeth, while others in the same row remain firm, it will be sufficient to close the mouth and apply a bandage as for fracture of the inferior maxilla; in others the teeth and their alveoli, ought to be fastened with silk, or gold or silver thread; or gold or silver clasps may be applied, or gutta percha moulded to the teeth and jaw.

In a case of fracture of the right superior maxilla reported by Baker, of Norwich, N. Y., complicated with a fracture of the inferior maxilla, the alveoli were retained in place very perfectly by a mould of gutta percha.‡ Neill, of Philadelphia, has also reported three cases of fracture of the bones of the face, involving the superior maxilla, in two of which the eyes were made to protrude more or less from their sockets.|| The loosened alveoli were made

---

* *Op. cit.*, vol. i, p. 376. Paris ed.

† News letter, April, 1854. Also, Bost. Med. and Surg. Jour., vol. li, p. 246.

‡ New York Jour. of Med., vol. i, 3d ser., pp. 362.

|| See Observations first and second, under fractures of the malar bone; in which cases the orbital plate of the malar bone was pushed into the sockets.

fast by wire. The subsequent deformity was inconsiderable, yet in no instance was the restoration complete.* The same method was adopted successfully by a surgeon in Virginia, in the case of a negro 50 years old, where most of the teeth of the left upper jaw were forced into the mouth, carrying with them their corresponding alveolar processes. The teeth remained firm in their sockets, but the separation of the bone was complete, the fragment being held in place only by the mucous membrane of the mouth. On the eighth day the surgeon found that the negro had removed the wire, and also the cork from between his teeth, and the maxillary bandage; but the soft parts had already united and the bones showed no tendency to displacement. His recovery was speedy and it was accomplished without any farther treatment.†

* Phil. Med. Exam., vol. x, new series, pp. 455–8.

Amer. Med. Gazette, vol viii, new series, p. 106.

